# Finite Element Analysis of a New Pedicle Screw-Plate System for Minimally Invasive Transforaminal Lumbar Interbody Fusion

**DOI:** 10.1371/journal.pone.0144637

**Published:** 2015-12-09

**Authors:** Jie Li, Jin Shang, Yue Zhou, Changqing Li, Huan Liu

**Affiliations:** Department of Orthopedics, Xinqiao Hospital, The Third Military Medical University, Chongqing, China; University of Michigan, UNITED STATES

## Abstract

**Purpose:**

Minimally invasive transforaminal lumbar interbody fusion (MI-TLIF) is increasingly popular for the surgical treatment of degenerative lumbar disc diseases. The constructs intended for segmental stability are varied in MI-TLIF. We adopted finite element (FE) analysis to compare the stability after different construct fixations using interbody cage with posterior pedicle screw-rod or pedicle screw-plate instrumentation system.

**Methods:**

A L3–S1 FE model was modified to simulate decompression and fusion at L4–L5 segment. Fixation modes included unilateral plate (UP), unilateral rod (UR), bilateral plate (BP), bilateral rod (BR) and UP+UR fixation. The inferior surface of the S1 vertebra remained immobilized throughout the load simulation, and a bending moment of 7.5 Nm with 400N pre-load was applied on the L3 vertebra to recreate flexion, extension, lateral bending, and axial rotation. Range of motion (ROM) and Von Mises stress were evaluated for intact and instrumentation models in all loading planes.

**Results:**

All reconstructive conditions displayed decreased motion at L4–L5. The pedicle screw-plate system offered equal ROM to pedicle screw-rod system in unilateral or bilateral fixation modes respectively. Pedicle screw stresses for plate system were 2.2 times greater than those for rod system in left lateral bending under unilateral fixation. Stresses for plate were 3.1 times greater than those for rod in right axial rotation under bilateral fixation. Stresses on intervertebral graft for plate system were similar to rod system in unilateral and bilateral fixation modes respectively. Increased ROM and posterior instrumentation stresses were observed in all loading modes with unilateral fixation compared with bilateral fixation in both systems.

**Conclusions:**

Transforaminal lumbar interbody fusion augmentation with pedicle screw-plate system fixation increases fusion construct stability equally to the pedicle screw-rod system. Increased posterior instrumentation stresses are observed in all loading modes with plate fixation, and bilateral fixation could reduce stress concentration.

## Introduction

Transforaminal lumbar interbody fusion (TLIF) is used to achieve a solid arthrodesis of spinal segments which sustains loading, while maintaining disc space height, preserving foraminal dimensions, and restoring sagittal plane alignment. Paraspinal muscle dissection and retraction in open TLIF remains a drawback, which would cause muscle denervation, atrophy and consequently persistent lower back pain [1]. Because of the development of cannulation systems and percutaneous pedicle screw implantation techniques, MI-TLIF was first introduced by Foley et al. [2, 3] as an alternative technique to open TLIF to reduce soft-tissue damage. MI-TLIF was reported to be associated with less blood loss, shorter hospital stay, lower complication rate, similar high fusion rate and a trend toward better functional outcomes compared to open TLIF [4–6]. In MI-TLIF, facetectomy (unilateral or bilateral) and partial discectomy are involved, which causes surgical segment instability and needs to reconstruct immediate stability by using intervertebral graft and posterior instrumentations. Polyaxial pedicle screw fixation is used to restore lumbar lordosis and disc height, while an interbody cage is positioned under compression in the anterior or middle column. However, cage subsidence [7] and limit sagittal balance reconstruction [8] were reported in the use of polyaxial pedicle screw-rod system. Along with the development of modern surgical instrumentation, a new monoaxial pedicle screw-plate system has been introduced as an alternative to the polyaxial pedicle screw-rod system for MI-TLIF.

A finite element model has the advantages of easily modifying TLIF cage and posterior implant geometry to observe the altered load transfer on the individual motion segment, and to analyze the stress distribution on these constructs. A few studies have investigated the biomechanical performance of TLIF procedures with different instrumentation methods [9–11]. Different longitudinal connectors used in this new system (plate vs. rod) may alter the load transfer and affect stress distribution of the posterior instrumentation system. The purpose of this study was to analyze the kinematic and mechanical behaviour of the new plate system. We developed a finite element model of the intact L3-S1with L4/5 right sided facetectomy and discectomy. Range of motion in flexion, extension, lateral bending and axial rotation, Von Mises stress of posterior implant and interbody graft were evaluated.

## Materials and Methods

We developed a three-dimensional nonlinear finite element model of the lumbosacral spine (L3-S1), which consisted of four vertebrae, three discs and associated spinal ligaments. The study was approved by the Ethics Committee of the Second Affiliated Hospital of Third Military Medical University, Chongqing, China. After obtaining the informed consent of a volunteer, geometrical details of bony structures were obtained from 64 spiral computed tomography (CT) images of a 29-year-old healthy male (70kg and 175cm) without history of spine injury, osteoporosis and radiographic evidence of degenerative sign. The PC used in this study was Think Station D20 with Xeon X5650 CPU and 16 GB RAM

### Geometrical Reconstruction

The CT scans of the L3-S1 were obtained at a 0.5-mm interval and 0.6-mm resolution using CT scanner (Brilliance iCT, Philips, the Netherlands). The image were imported into medical image-processing software (Mimics 10.0, Materialise Inc., Belgium) to reconstruct the geometric surface of the lumbosacral vertebrae. The surface model was then exported into Geomagic Studio 2012 (Geomagic Inc., USA) to enhance the quality of the surface. We use the software to perform smoothing process, remove the high aspect ratio elements and repair gaps left in the surface model. The solid model was generated by SolidWorks 2012 (Dassault Systèmes SolidWorks Corp., France) from the geometric data. Eventually, it was imported to finite element software (Abaqus 6.9.1, Dassault Systemes Simulia Corp., Providence, RI, USA) for meshing and analyzing.

**Fig 1:**
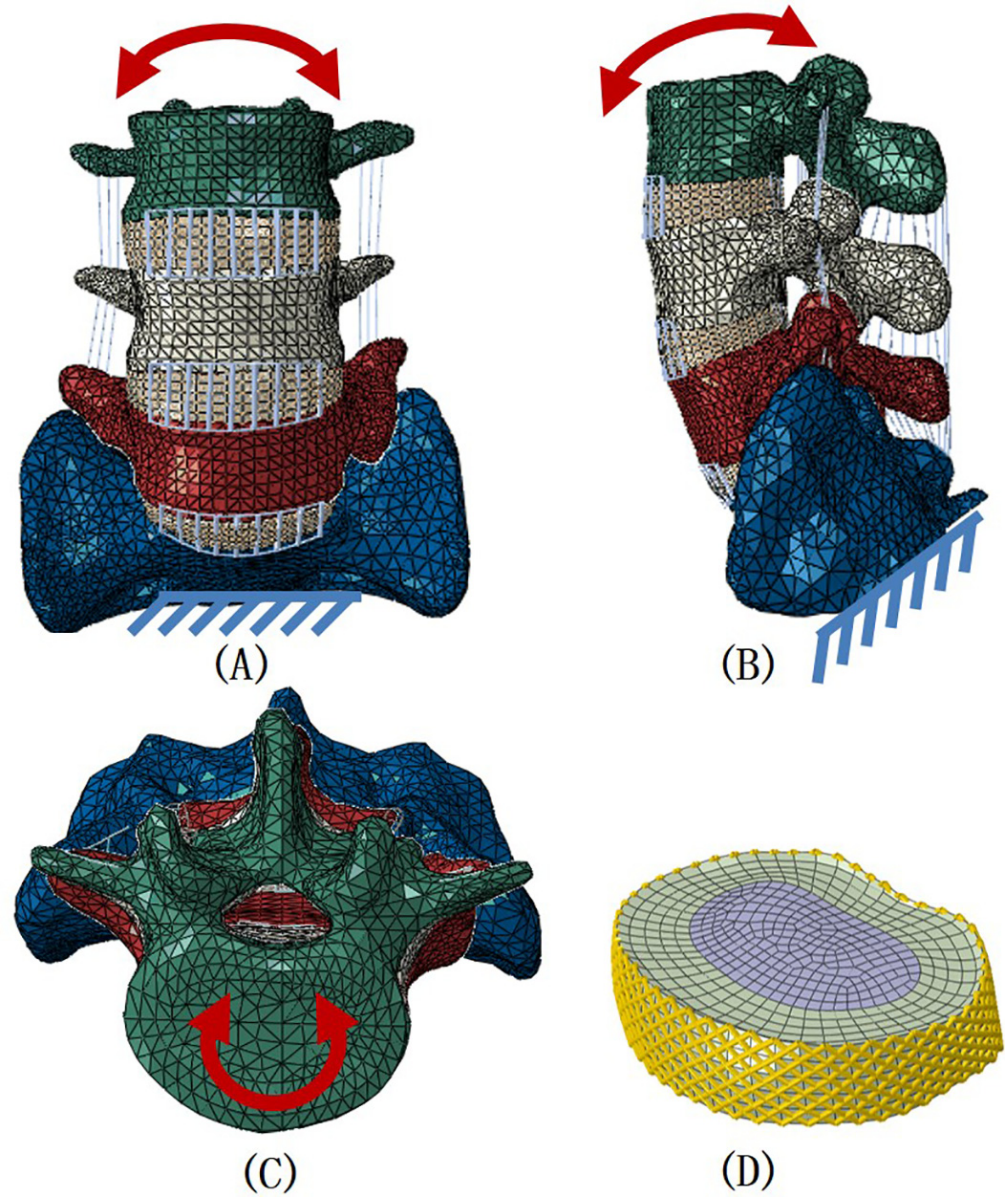
The intact model: sectioned in the coronal (A), transverse (B), and sagittal planes(C)), with loading and boundary conditions. The disc showing the inclination of the fibers (part D).

The vertebrae were composed of a solid volume (cancellous bone) and a layer of shell (cortical bone and endplate) with a thickness of 0.5 mm [12]. The intervertebral disc was constructed as a continuum structure occupying the intervertebral space. Each disc was composed of incompressible nucleus pulposus and annulus fibrosus, and the ratio was approximately 4-to-6 [13, 15]. The fluid-like behavior of the incompressible nucleus pulposus was simulated with linear elasticity (E = 0.1Mpa, Poisson’s Value = 0.49). A layer of netlike annulus fibers was adhered on the circumferential surface of the substance, with an inclination between 15° and 45° with respect to the transverse plane [14–16]. The fiber was assigned with 19% volume of the annulus fibrosus [14, 16]. Intervertebral ligaments include anterior longitudinal ligament, capsular ligament, posterior longitudinal ligament, flaval ligament, and interspinous and supraspinous ligament. These ligaments were modeled in trusses with anatomical insertion sites. The lumbosacral spine and the components were presented in Fig 1.

### Material Property Assignment and Mesh

The metal component and cage were made of Ti6Al4V and polyetheretherketone, respectively. The material properties and mesh type of lumbosacral components and implants were listed in Table 1. The element nodes of the cortical bone and endplate coincided precisely with the nodes of the cancellous bone, whereas the nodes of the annulus fibers coincided with the matrix of the annulus ground substance. In the healthy lumbosacral spine, the total numbers of nodes and elements were 68, 868 and 154,967, respectively. The numbers of S3, C3D4, C3D8R and T3D2 element type were 13,959, 104,693, 33,291, and 3,024, respectively.

**Table 1:** Material property and mesh type of spinal components and implants.

Component	Yong’s modulus (MPa)	Poisson’s ratio	Element type	Cross section (mm^2^)
Cortical bone [13]	12000	0.30	Triangle shell (S3)	
Endplate [16]	1200	0.29	Triangle shell (S3)	
Cancellous bone [13]	100	0.30	Tetrahedron (C3D4)	
Annulus ground substance [13]	4.2	0.45	Hexahedron (C3D8R)	
Nucleus pulposus [13]	0.1	0.49	Hexahedron (C3D8R)	
Annulus fiber [16, 17]	450	0.45	Truss (T3D2)	
Pedicle screws and rod/plate[9]	110,000	0.30	Hexahedron (C3D8R)	
PEEK cage[9]	3600	0.30	Tetrahedron (C3D4)	
Bone graft[12]	12000	0.30	Hexahedron (C3D8R)	
Ligaments [17]				
Anterior longitudinal ligaments	7.8	0.30	Truss (T3D2)	63.7
Posterior longitudinal ligaments	10	0.30	Truss (T3D)	20.0
Supraspinous ligament	8	0.30	Truss (T3D2)	30.0
Interspinous ligament	10	0.30	Truss (T3D2)	40.0
Ligamentum flavum	15	0.30	Truss (T3D2)	40.0
Intertransverse ligament	10	0.30	Truss (T3D2)	1.80
Capsular ligament	7.5	0.30	Truss (T3D2)	30.0

### TLIF with Instrumentation Models

**Fig 2:**
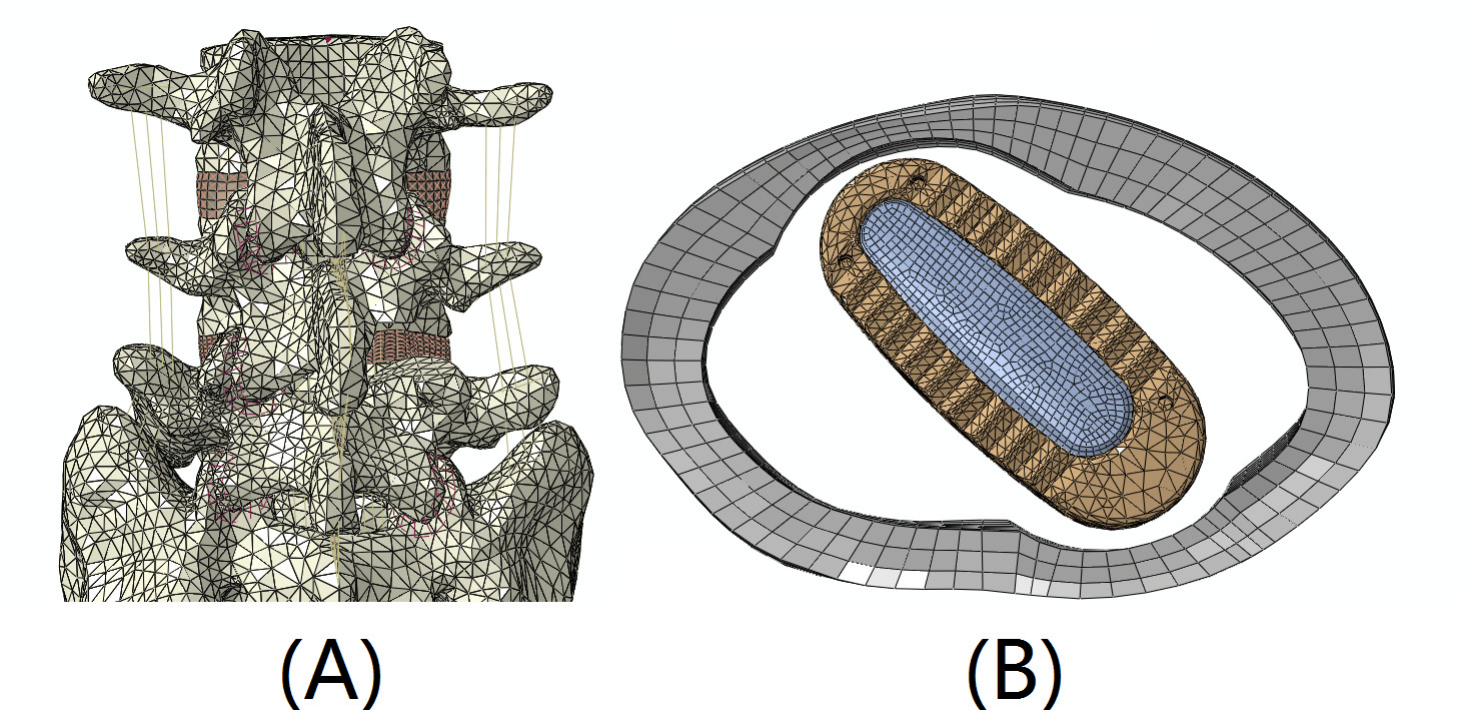
Transforaminal lumbar interbody fusion model. (A) L4-5 right side facetectomy, (B) PEEK cage and bone graft was placed after partial discectomy.

The FE model was modified to simulate the MI-TLIF surgical procedure at the L4–L5 level via a transforaminal approach by the application of facetectomy and partial discectomy [9]. The nucleus was totally removed and the two inner layers of annulus were removed at L4-5 segment. One polyetheretherketone cage filled with bone graft was placed in the disc (Fig 2). We used intervertebral graft to describe the cage and cortical bone inside it. The simulated standard cage (10 mm height) was boxed-shaped, with blunt tooth on superior and inferior surfaces.

**Fig 3:**
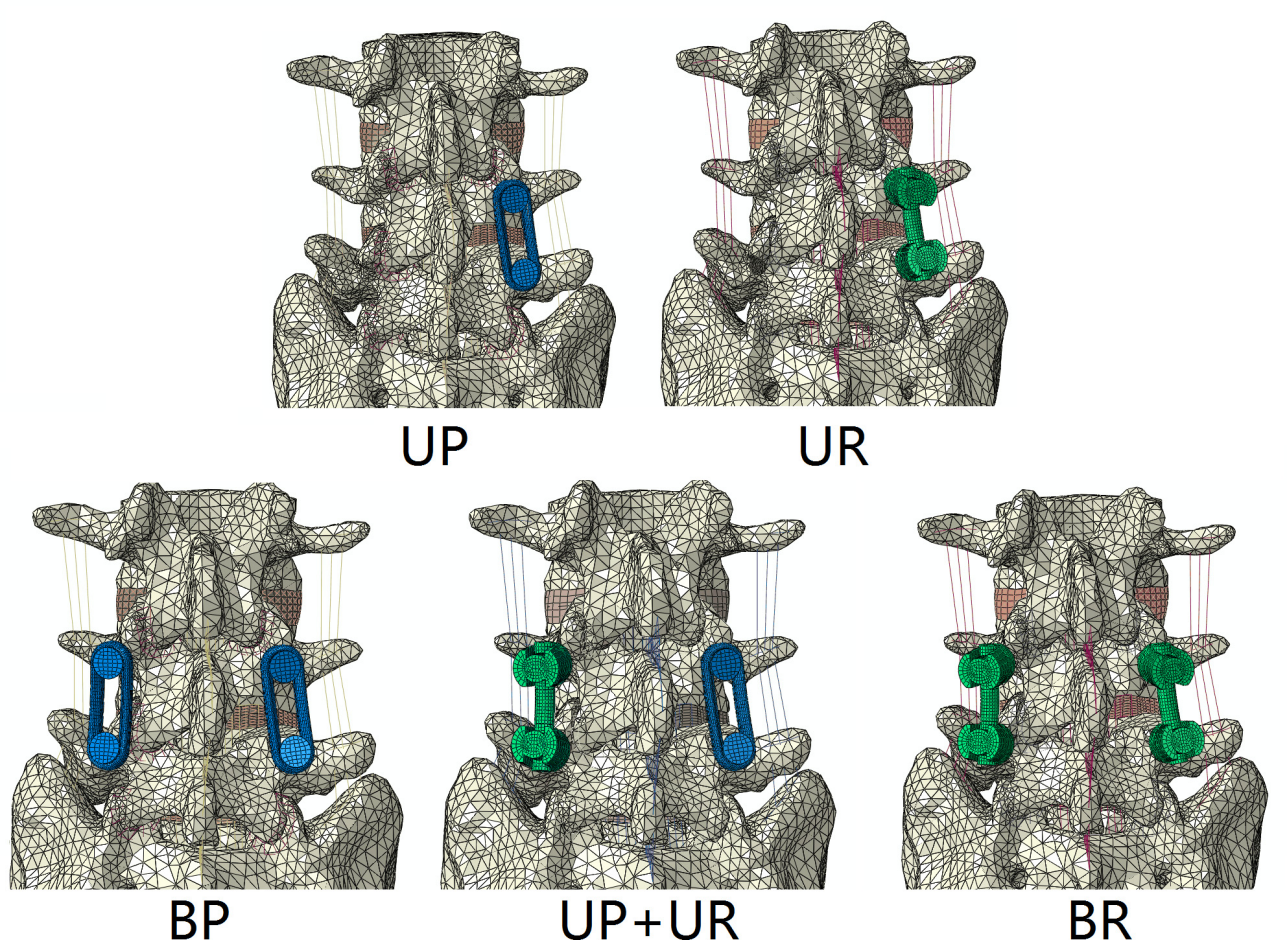
Finite element model of fixation modes. Five finite element models with L3-S1 after right sided TLIF. UP, unilateral pedicle screw-plate fixation; UR, unilateral pedicle screw-rod fixation; BP, bilateral pedicle screw-plate fixation; BR, bilateral pedicle screw-rod fixation; UP+UR, unilateral pedicle screw-plate supplemented with pedicle screw-rod fixation.

Five instrumentation models were established using the TLIF model (Fig 3): unilateral pedicle screw-plate fixation (UP), unilateral pedicle screw-rod fixation (UR), bilateral pedicle screw-plate fixation (BP), bilateral pedicle screw-rod fixation (BR), pedicle screw-plate supplemented with pedicle screw-rod fixation (UP+UR). The right side from posterior coronal view of the model was assigned to undergo instrumentation in unilateral fixation. The posterior instrumentation consisted of pedicle screw (55mm long, 6.5mm diameter) and longitudinal connector (rod: 45mm long, 5.5 mm diameter/plate: 45mm long, 5.5mm height, 11mm wide) spanning between adjacent screws.

### Contact Definitions and Boundary Conditions

The insertion points of the ligaments and the interfaces of the intervertebral disc were attached onto the vertebrae, whereas the interaction between the facet joint was assigned with frictionless sliding contact formulation [14–16].The bone and pedicle screw interface and pedicle screw and rod/plate interface were assigned with a “tie” constraint. A finite sliding algorithm with a coefficient of friction of 0.2 was defined between the cage and end plate to allow for any small relative displacements between the two contacting surfaces [9], to simulate the early postoperative stage after spinal instrumentation. The articulating facet joints were modeled using surface to surface contact elements.

Two types of loading conditions corresponding to loads were used for model validation and model predictions. For four pure moments, the results of a range of motion were compared with those of previous in vitro studies by Dahl et al. [18] and Soriano-Baron et al. [19]. The nodes of the inferior surface of S1 was constrained in all degrees of freedom. We applied the same loading conditions as Dahl et al [18] (8 Nm in all motions in a stepwise manner) and Soriano-Baron et al [19] (7.5 Nm in all motions in a stepwise manner). The loads were imposed to the reference node on the top surface of L3. The ROM of segment of FE model was compared with that of the cadaveric model. The second type of loading condition was the load control protocol for model prediction. This load control protocol was stepwise and involved the application of a 7.5 Nm flexion, extension, torsion, lateral bending pure moment to test models on the L3 vertebral body with a 400 N follower pre-load [20, 21]. We set a reference point on the anterior wall of each vertebral body. Under various loading conditions, the angular dislocations of each reference point were outputted and the subtraction of angular dislocations between two adjacent vertebral bodies was ROM.

We followed the convergence test method referred by Womack et al. [22] and created four models of varied mesh resolution to verify the model convergence. Additionally, mesh density was elevated by three folds between the coarsest and finest models (from 102,455 meshes to 307,689 meshes). Model convergence was tested throughout a 7.5 Nm with 400 N following pre-load on L3. Convergence of average largest Von Mises stresses of L4/5 nucleus, segmental ROM of each level and total ROM were tested. Convergence within 3% on all the parameters was obtained to ensure that the results were not relevant to the mesh density.

## Results

### Model Validation

**Fig 4:**
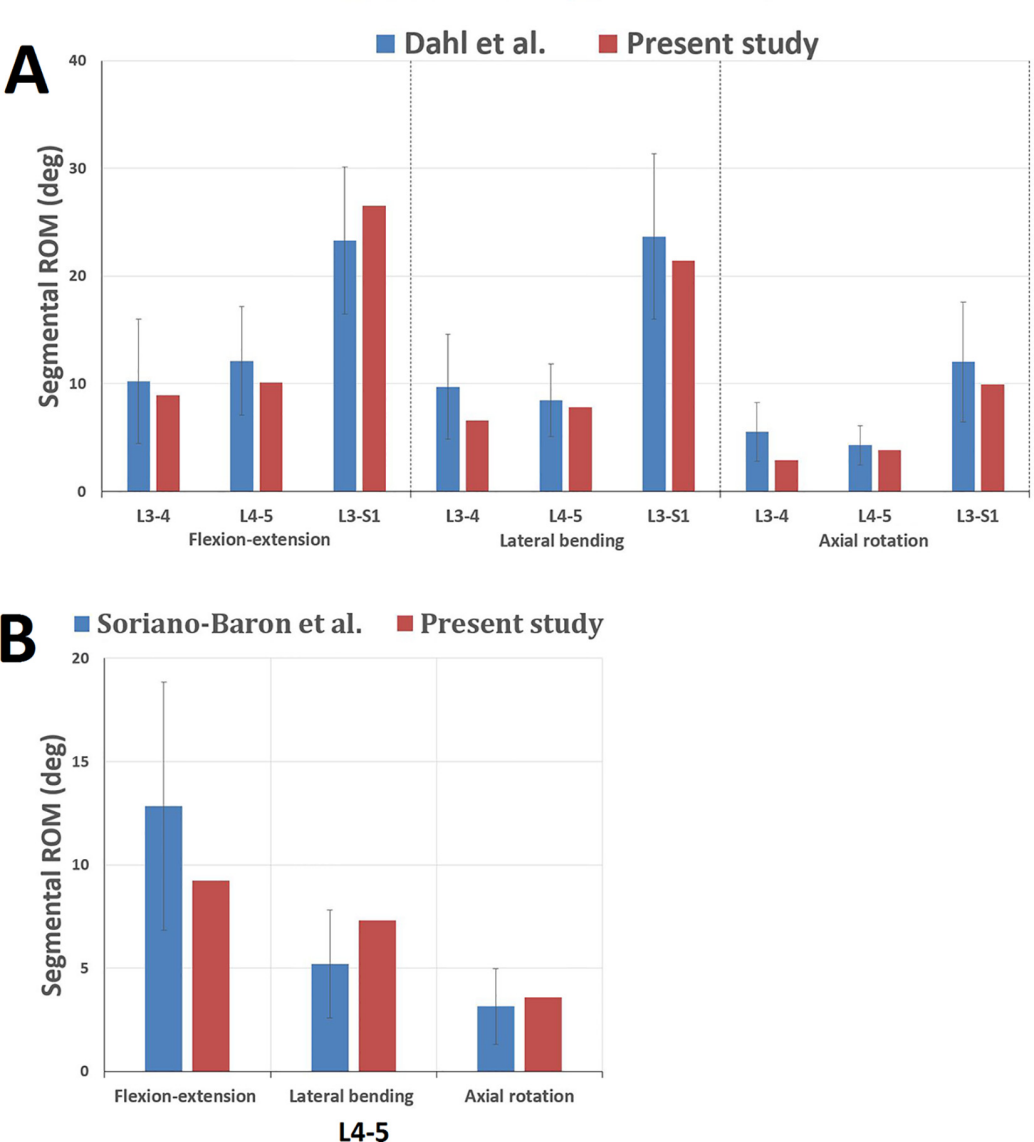
Comparison between the current intact model and previous studies for the validation. A. Comparison with Dahl et al. B. Comparison with Soriano-Baron et al.

The intact model was used for the validation and the results of the range of motion were compared with those of previous in vitro studies by Dahl et al. [18] and Soriano-Baron et al. [19]. All of the data in this study were within plus/minus 1 standard deviation of the average reported by the previous study (Fig 4). Therefore, the models in the present study were effective for further analyses.

### Range of Motion

**Fig 5:**
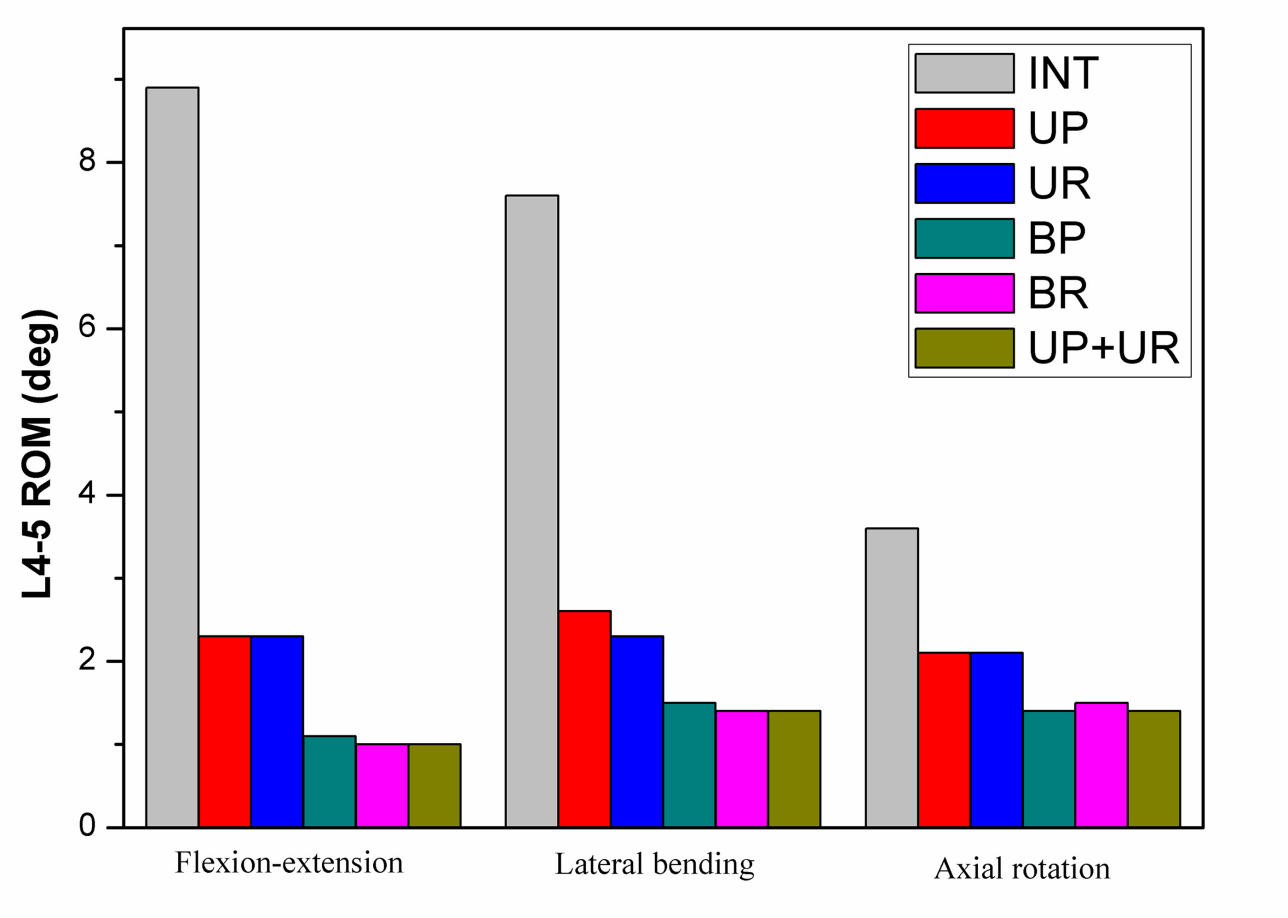
Range of motion values at L4-L5 in intact and instrumentation models.

The range of motion (ROM) of the L4-5 segment was reduced for all construction techniques compared with that in the intact condition (Fig 5). There were small differences in the ROM observed between the plate system and the rod system fixation, neither in unilateral fixation group nor in bilateral fixation group. Variation in ROM was noted with unilateral fixation when compared with bilateral fixation. ROM was reduced by 74% and 88% in flexion-extension, 66% and 80% in lateral bending, 42% and 61% in axial rotation of the intact ROM, for unilateral and bilateral plate fixation respectively.

### Posterior Instrumentation Stress Analysis

#### Von Mises Stress of the Pedicle Screw

**Fig 6:**
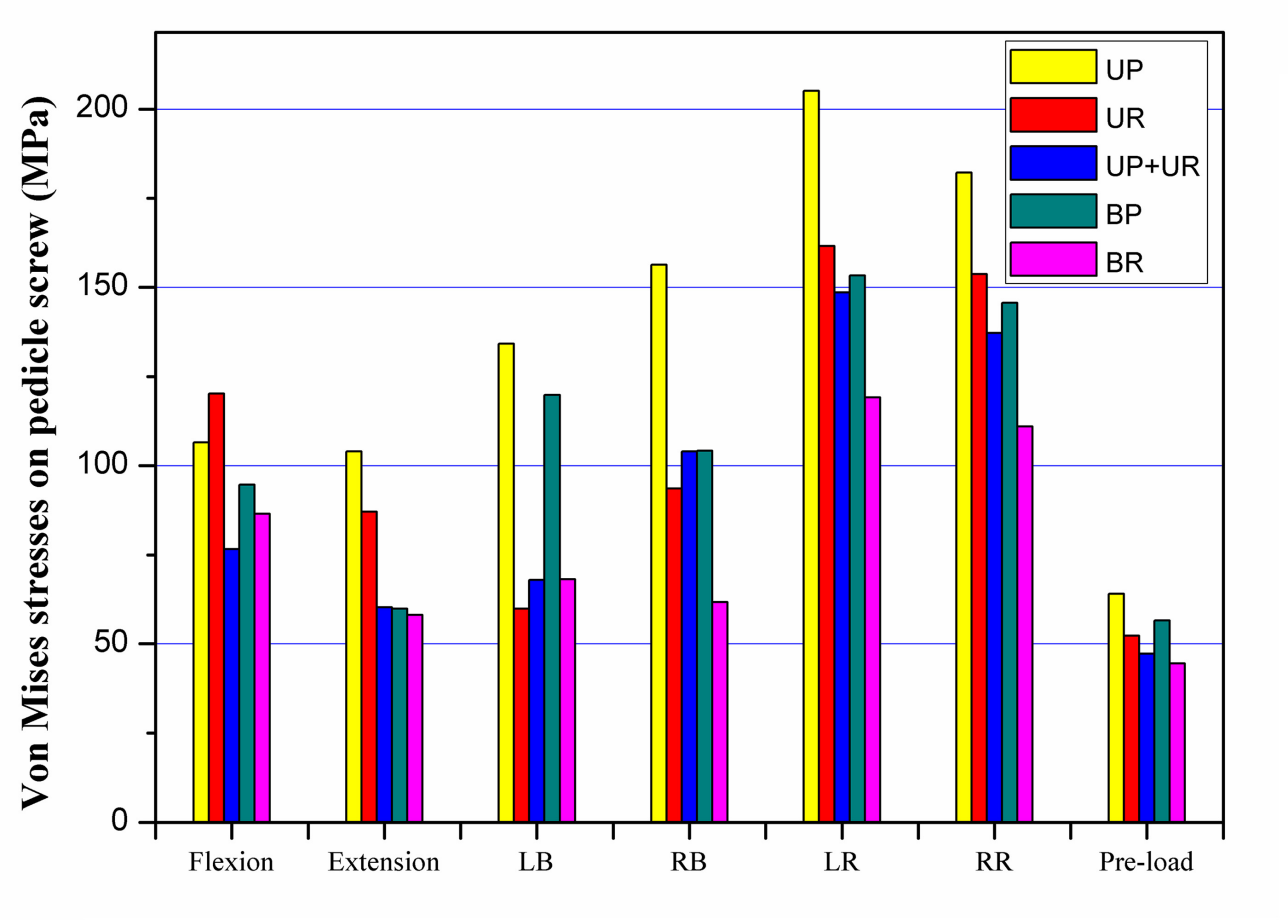
Maximum Von Mises stresses on the pedicle screw. LB: left lateral bending; RB: right lateral bending; LR: left axial rotation; RR: right axial rotation.

The largest maximal stresses of pedicle screws were all detected in axial rotation among all the fixation models during all states of motion (Fig 6). For unilateral fixation, the maximum stresses calculated for the caudal screws ranged from 0.9 to 2.2 times higher for plate system compared with rod system. The largest peak stresses in the plate system and the rod system were 205.1 MPa and 161.6 MPa in left axial rotation, respectively. The peak stresses in the plate system were 2.2 times higher than those in the rod system in left lateral bending, and 0.9 times less than those for rod system in flexion. For bilateral fixation, there was little difference in peak stresses between the plate system and the rod system in flexion and extension. The largest peak stresses in the plate system and the rod system were 153.3 MPa and 119.1 MPa in left axial rotation, respectively. The largest difference was noted in left lateral bending, in which the peak stress of the plate system was 1.8 times higher than that of rod system. Comparing the maximum stresses between the unilateral and bilateral fixation by the plate system, the maximum stresses ranged from 1.1 to 1.7 times higher for unilateral fixation compared with bilateral fixation. The peak stresses in unilateral rod fixation were 0.9 to 1.5 times higher than those for bilateral rod fixation. The largest peak stress was 148.6 MPa in UP+UR fixation modes in left axial rotation.

#### Von Mises Stress of the Rod/Plate

**Fig 7:**
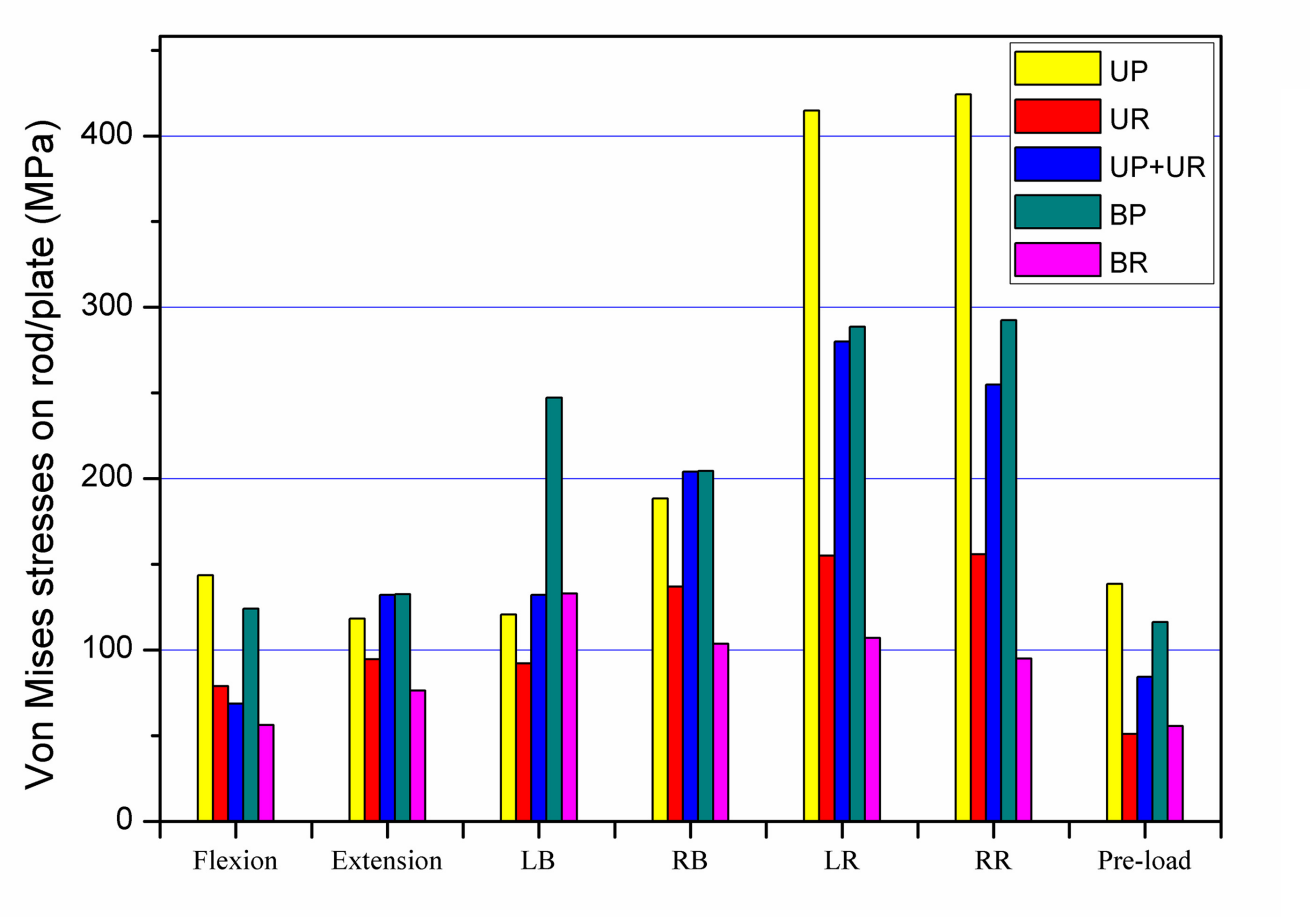
Maximum Von Mises stresses on the longitudinal connector (rod/plate). LB: left lateral bending; RB: right lateral bending; LR: left axial rotation; RR: right axial rotation.

**Fig 8:**
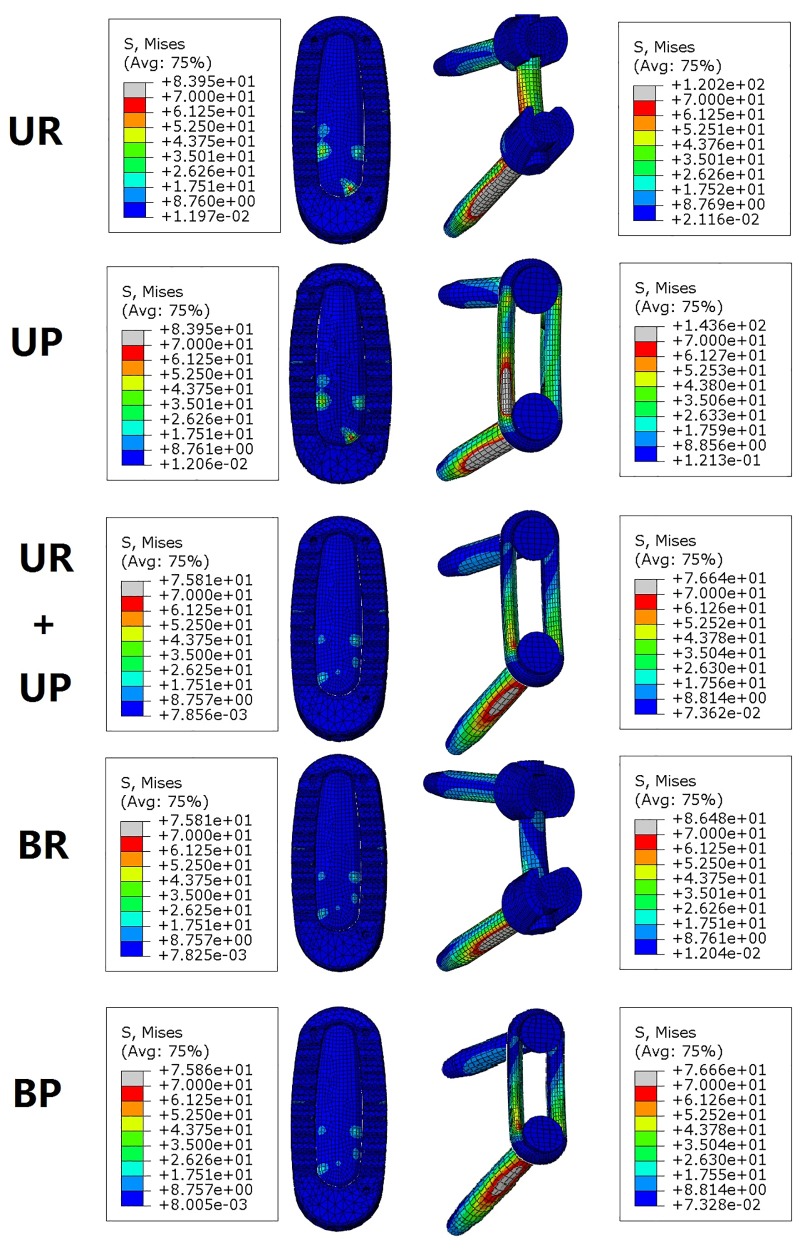
Stress nephogram of the intervertebral graft and posterior instrumentation on the right side under flexion. Grey color region indicated the over 70 MPa Von Mises stresses distribution. Cage and bone graft were showed on the left and the posterior instrumentations were showed on the right.

The largest maximal stresses of longitudinal connector were all detected in axial rotation among all the fixation models during all states motion (Fig 7). For unilateral fixation, the maximum stresses calculated for the longitudinal connector ranged from 1.3 to 2.7 times higher for plate system compared with rod system. The largest peak stresses in the plate system and the rod system were 424.3 MPa and 155.9 MPa in right axial rotation, respectively. The peak stresses in the plate system were 2.7 times higher than those in the rod system in axial rotation, and 1.3 times higher than those in rod system in extension. For bilateral fixation, the maximum stresses ranged from 1.7 to 3.1 times higher for plate system compared with rod system. The largest peak stresses in the plate system was 292.6 MPa in right axial rotation while in the rod system was 132.9 MPa in left lateral bending. The peak stresses in the plate system were 3.1 times higher than those in the rod system in axial rotation, and 1.3 times higher than those for rod system in extension. Comparing the maximum stresses in the unilateral and bilateral fixation of the plate system, the maximum stresses ranged from 0.5 to 1.5 times higher for unilateral fixation compared with bilateral fixation. The peak stresses in unilateral rod fixation were 0.7 to 1.6 times than those in bilateral rod fixation. The peak stresses in bilateral fixation by the plate or rod system were less than those for the unilateral fixation in left lateral bending. The largest peak stress was 280.1 MPa in UP+UR fixation modes in left axial rotation. The gray color area on the nephogram (Fig 8) showed the site of von Mises stresses over 70 MPa on the posterior instrumentation under flexion motion. Larger areas of over 70 MPa posterior instrumentation stresses were observed with unilateral fixation.

### Intervertebral Graft Stress Analysis

**Fig 9:**
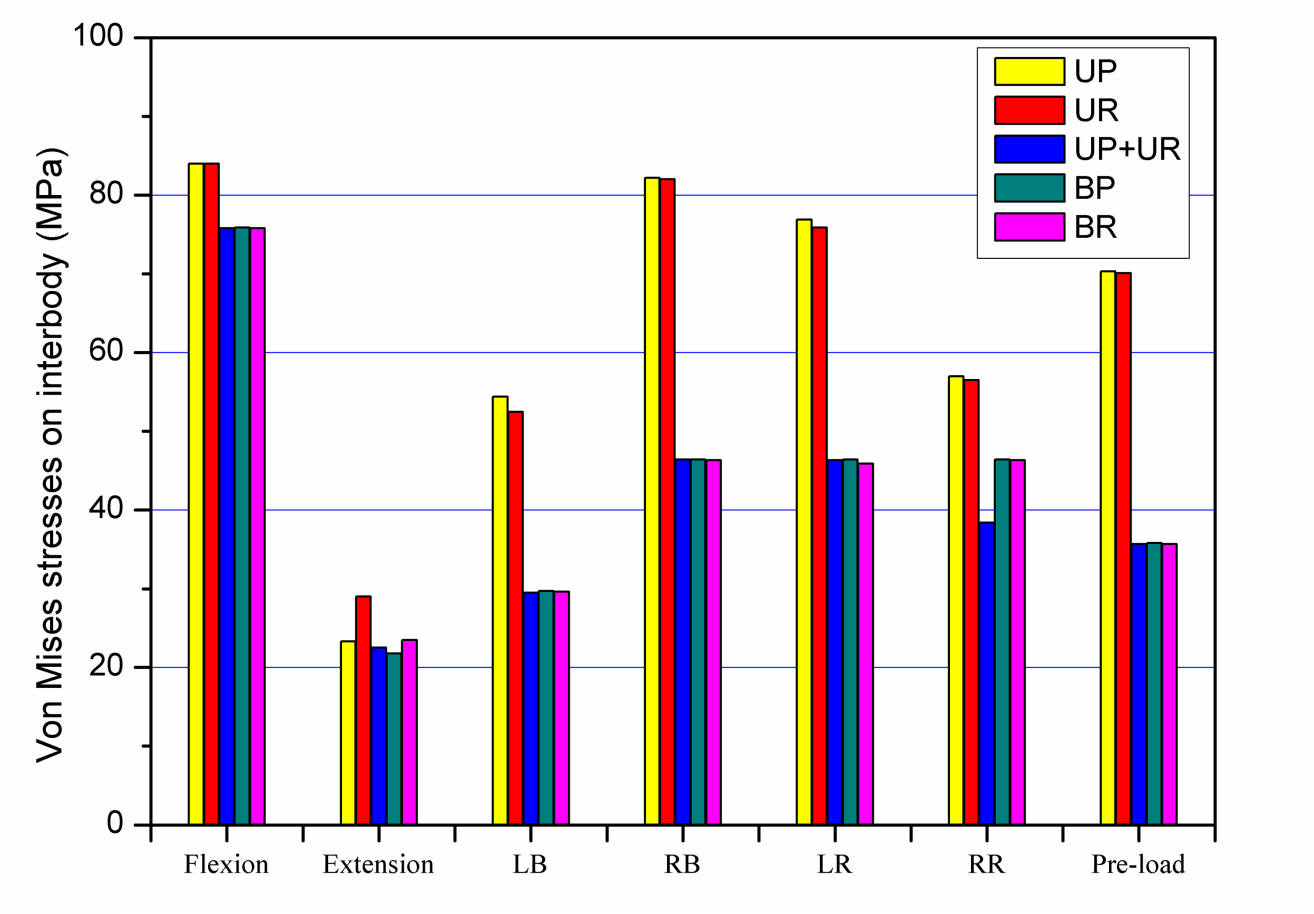
Maximum Von Mises stresses on intervertebral graft. LB: left lateral bending; RB: right lateral bending; LR: left axial rotation; RR: right axial rotation.

Von Mises stresses on the graft obtained for each construct were showed in Fig 9. For unilateral posterior fixations and bilateral posterior fixations, the largest peak stresses calculated were 84 MPa and 75.8 MPa in flexion motion, respectively. Comparing the stresses in the unilateral and bilateral fixation of the plate system, the ratio ranged from 1.1 to 2.0. Similarly in the rod system, the stresses on intervertebral graft in unilateral rod fixation was 1.1 to 2.0 times higher compared with those in bilateral rod fixation.

## Discussion

The old plate system had been gradually replaced by the rod system [23]. The pedicle screw and plate system initially evolved from the plate system designed for limb fracture fixation, but in terms of spinal fixation, the locations of screw holes strongly hindered the proper positioning of pedicle screw and plate [24]. In addition, there is a lack of an interface between the screw and the plate that allowed the construct angle to be adjusted in situ and subsequently rigidly locked [25]. The previous plate systems were also less rigid than the screw-rod systems because of the weakness at the screw-plate interface and their thinner longitudinal cross section [23]. The new pedicle screw and plate system attempts to address some of these limitations: the accommodation angle within 15 degrees, the slotted plate, the use of monoaxial pedicle screw and a special guide system for minimally invasive system. Moreover, the old plate system had mostly been used in posterolateral fusion, in which the load transduction made its structural defect more incompetent. Nowadays, the transformational interbody fusion is prevalent and the load transduction is different from that in posterolateral fusion. The load transduction in posterolateral fusion mainly depends on the posterior instrumentation constructs, while in transforaminal interbody fusion the load is shared by the interbody cage and the posterior instrumentation constructs. This difference results in decreased stiffness demands for posterior instrumentation constructs in transforaminal interbody fusion. Therefore the experience of using the older plate system is not sufficient for us to predict the effects of the novel plate system in single-level MI-TLIF.

Additionally, the pedicle screw-rod system used in MI-TLIF is imperfect. The polyaxial pedicle screw was used in the rod system in MI-TLIF and its ball-in-cup structure significantly reduced the strength of the whole assembly compared with that of the monoaxial pedicle screw [26–28]. This would lead to a decrease in yield strength in static tests and increase cage subsidence risk. In the meantime, Kuklo et al [29] showed that the rotational stability was weak in the unilateral rod fixation in a biomechanical study. Huang et al [30] reported the decreased rotation torque in the round rod compared with that in the polygonal rod in cantilevered pedicel screw system. When the polyaxial pedicle screw-rod system is used in MI-TLIF, it is difficult to achieve effective reduction of sliding vertebra or distraction-compression procedure. The ball-in-cup structure separated the screw body from the outside tools and the correction force was not able to reach the anterior column of vertebrae, while the monoaxial pedicle screw could achieve better correction performance.

We conducted the FE analysis to evaluate the stability of the five constructs under different loading conditions, assessing motion at the fixation level and estimating the stresses experienced by the posterior instrumentation and intervertebral graft. After performing simulations of the right side facetectomy and partial discectomy through a transforaminal approach, we found that all reconstructive configurations provided a substantial reduction in ROM compared with the intact lumbar spine. The pedicle screw-plate system fixation reduced ROM at the L4-L5 between 61%-88% compared with intact conditions in all loading modes, consistent with the results from previous studies [9–11]. In contrast, the pedicle screw-rod fixation reduced ROM between58%-89%, which was similar to the plate system. The stresses on the intervertebral graft for the plate system and the rod system were very close to each other in all loading modes. Our findings about the ROM and the stresses distribution on the intervertebral graft indicated that the plate system and the rod system provided similar fixation effect at the fusion level. However, the stresses on the posterior instrumentation system were quite different. The peak stresses of the posterior construct were greater for the plate system compared with those for the rod system in most of the loading modes, with maximal differences observed during axial rotation and left lateral bending. We combined two parts of the results and got the conclusion that the plate system suffered higher stresses than the rod system in order to achieve the same fixation effect, which means the plate system was under a higher risk of implant failure.

The cross section area of the paired parallel bars on plate was designed to be the same with that of the single rod, so each bar with half of the rod cross section area suffered higher stresses than single rod under the same load. Biomechanical study indicated the decrease of the rotation torque in the round rod compared with the in the polygonal rod in cantilevered pedicel screw system [30]. The plate system designed with a higher torsion inertia moment could apply more rotation stability theoretically, while the peak Von Mises stresses was 3.1 times greater than those for the rod system in axial rotation in this study. The biomechanical study indicated why the older plate system experienced higher construct failure rate in posterolateral fusion surgery without load sharing from the intervertebral graft. In fact, it remains unclear whether the calculated stresses on the plate system are exceeding its yield strength and could trigger structure failure in MI-TLIF. However, further biomechanical studies are required to ascertain whether the plate system will have a higher possibility of implant failure.

In this study, we also compared the fixation effect of the unilateral fixation and the bilateral fixation. Firstly, variation in ROM was noted with unilateral fixation when compared with bilateral fixation. Simultaneously, the stresses on intervertebral graft in unilateral fixation were larger compared with bilateral fixation. The distribution was eccentric under the axial force produced by flexion and pre-load modes. Moreover, the stresses on the posterior instrumentation were much larger on rod/plate and screws in unilateral fixation compared with the bilateral fixation. These results were consist with the previous studies about unilateral and bilateral posterior instrumentation in TLIF [9, 10]. Goel.et al. [31] have found that unilateral constructs that presented coupled motions due to the inherent asymmetry may fail to provide enough stability after complete disc decompression. Unilateral pedicle screw fixation may also provide only 50% of the stiffness of bilateral fixation, especially in rotational motion [32]. Chen SH et al. [10] confirmed that TLIF surgery didn’t favor implanting a diagonal cage and UPSF, as ROM increased and stresses concentrated on the neighboring annulus, cage-vertebral interface, and pedicle screw in contralateral axial rotation and lateral bending. Although the biomechanical analysis showed the unsatisfactory performance in the unilateral pedicle fixation, the clinical study results were more favorable. As noted previously, one-level unilateral pedicle screw instrumented MI-TLIF provided similar radiological and clinical outcomes compared to bilateral pedicle screw instrumented MI-TLIF [33–35].

There are a few limitations to this study. The most commonly used pedicle screw in minimally invasive spine surgery is polyaxial designed with ball-in-cup construct. It is different from the monoaxial pedicle screw in the yield load of screw-rod assembly [26]. We built the ball-in-cup construct of pedicle screw and used the bond relationship to set the “ball and cup” construct, assuming that there would be no relative rotational slip between the ball and cup construct after locked by set screws under various load motion. In fact, relative rotational slip of pedicle screw does exit in the clinical use, which will significantly affect the fixation effect. Moreover, as for most FE models, the results are limited by ignorance of the contribution of the musculature [9]. In addition, in line with other FE studies, the model in this study was reconstructed from a single person's image data and absent of any statistical analysis [15]. The image data may not be a typical case representing the mean level of a population. In a future study, we plan to use a larger cohort and build standard healthy spine models from the image data of healthy individuals. It will cost much more manpower, material resources and financial resources to carry out this plan.

## Conclusions

For single-level MI-TLIF procedures, posterior fixation using the new pedicle screw-plate system offers immediate stability and is equivalent to fixation using a pedicle screw-rod system, regardless of unilateral fixation mode or bilateral fixation mode. Moreover, augmentation with bilateral posterior fixation offers greater immediate stability compared with unilateral posterior fixation. Increased posterior instrumentation peak stresses are observed in all loading modes with plate fixation, and bilateral fixation could reduce peak stresses.

## Supporting Information

S1 Fig Stress nephogram of the instrumentation on the right side under loading conditions. Red color region indicated the over 50.0 MPa Von Mises stresses distribution.
(TIF)

S2 Fig Stress nephogram of the intervertebral graft. Red color region indicated the over 1.0 MPa Von Mises stresses distribution.
(TIF)

S3 Fig Models with varied mesh resolution. Model 1 with 102,455 meshes, Model 2 with 154,976 meshes, Model 3 with 200,581 meshes and Model 4 with 307,689 meshes.
(TIF)

S4 Fig Maximum Von Mises stresses in nucleus of L4/5. The difference between models was within 2.5%.
(TIF)

S5 Fig Range of motion values at each segment. The difference between models was within 1.15%.
(TIF)

S6 Fig Total range of motion values at L3-S1. The difference between models was within 2.63%.
(TIF)
