# Supplementary figures and images for: Finite Element Analysis of a New Pedicle Screw-Plate System for Minimally Invasive Transforaminal Lumbar Interbody Fusion
Source: PLoS One. 2015 Dec 9;10(12):e0144637. doi: 10.1371/journal.pone.0144637 (PMC4674154; doi:10.1371/journal.pone.0144637)

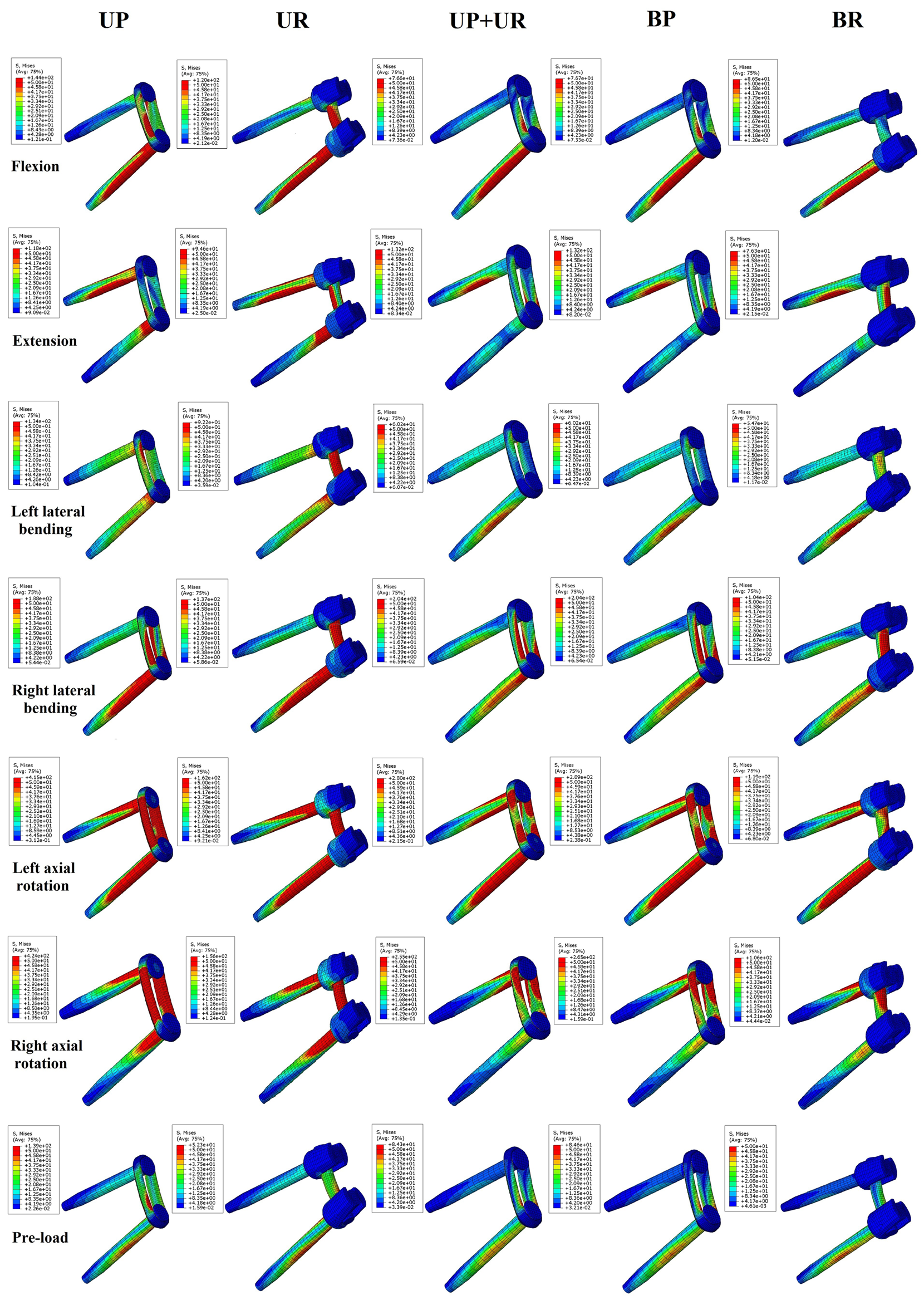

Supplement: S1 Fig — Red color region indicated the over 50.0 MPa Von Mises stresses distribution. (TIF) [file pone.0144637.s001.tif]

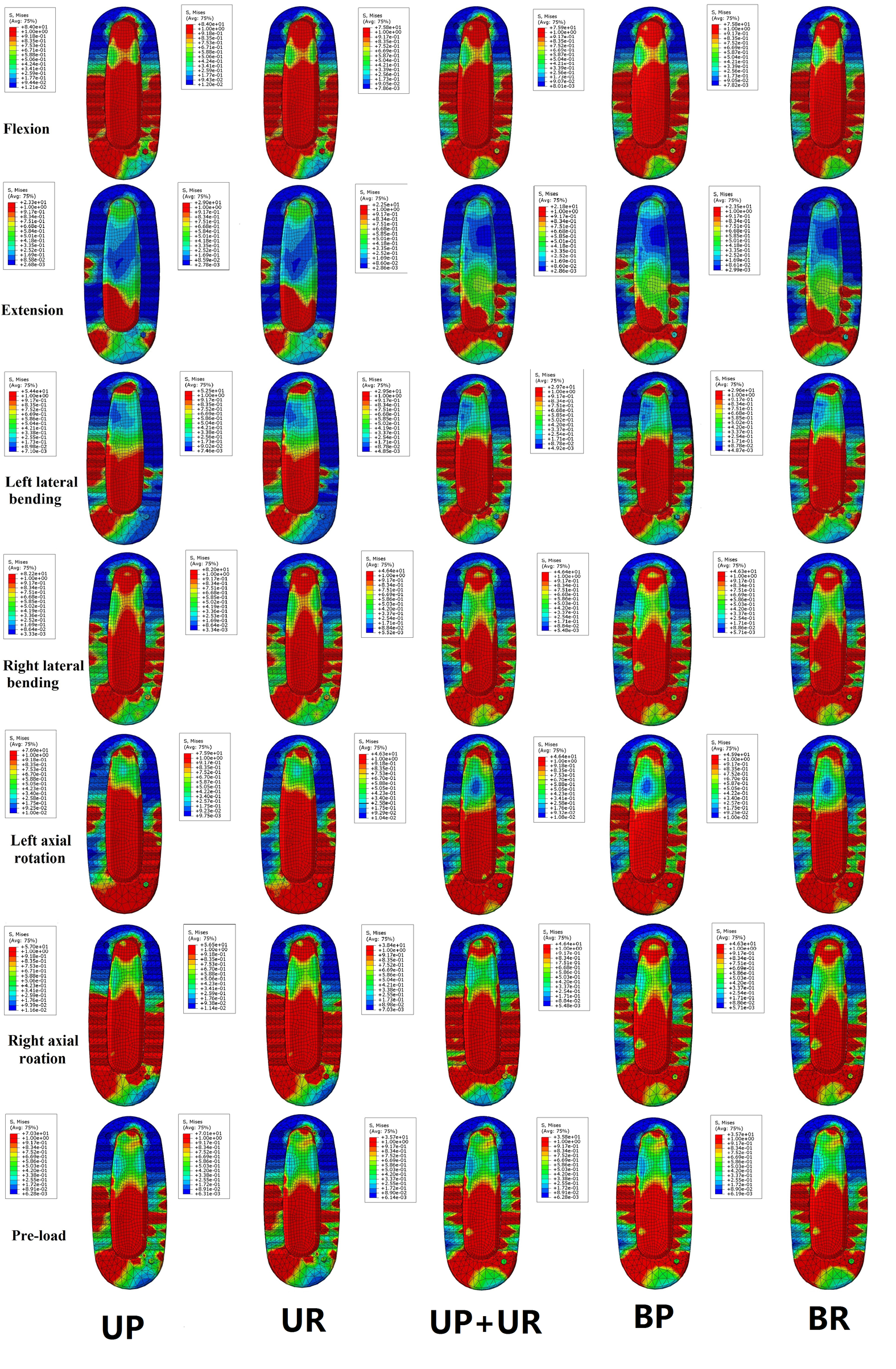

Supplement: S2 Fig — Red color region indicated the over 1.0 MPa Von Mises stresses distribution. (TIF) [file pone.0144637.s002.tif]

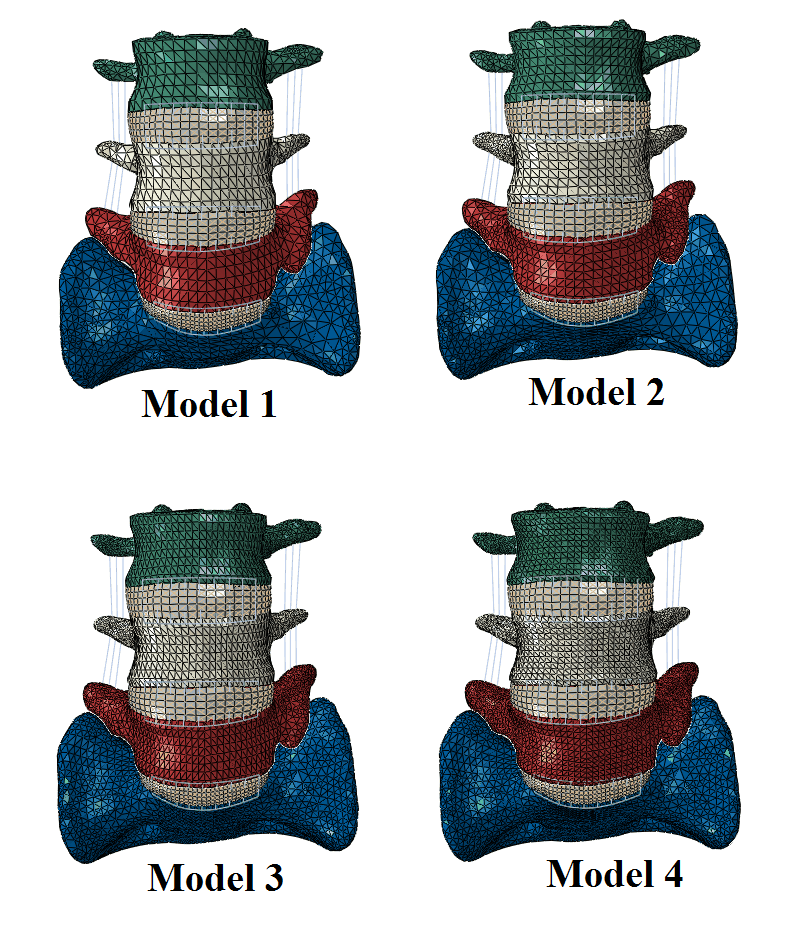

Supplement: S3 Fig — Model 1 with 102,455 meshes, Model 2 with 154,976 meshes, Model 3 with 200,581 meshes and Model 4 with 307,689 meshes. (TIF) [file pone.0144637.s003.TIF]

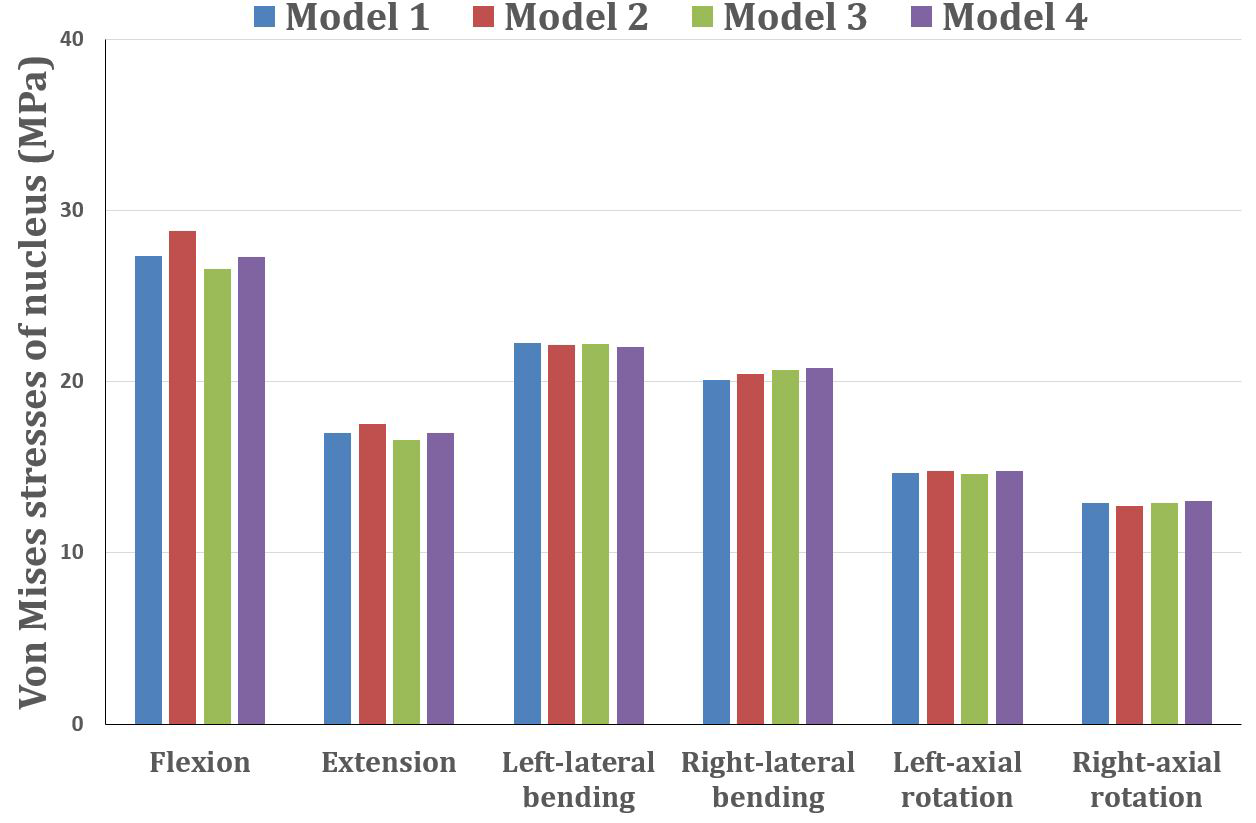

Supplement: S4 Fig — The difference between models was within 2.5%. (TIF) [file pone.0144637.s004.tif]

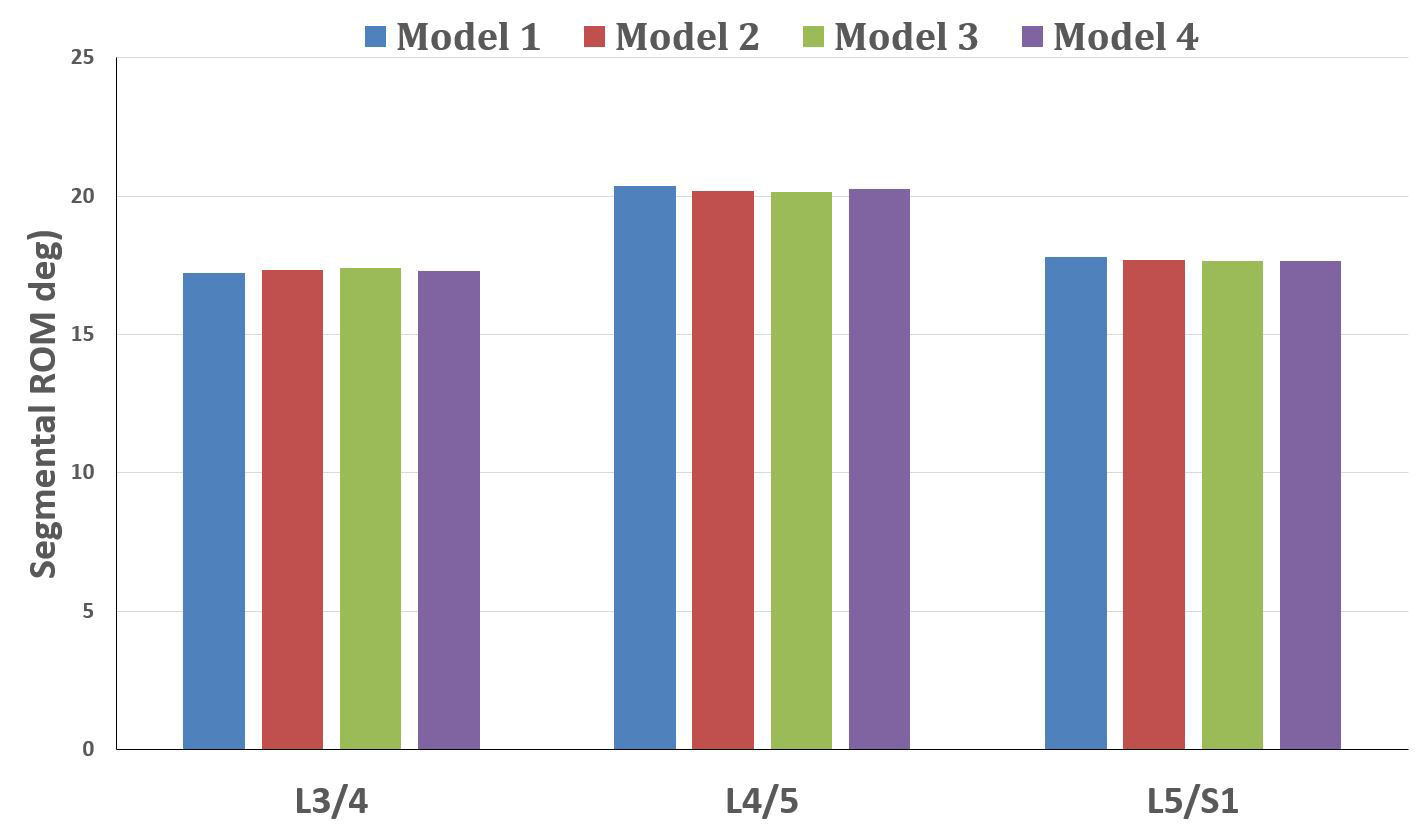

Supplement: S5 Fig — The difference between models was within 1.15%. (TIF) [file pone.0144637.s005.tif]

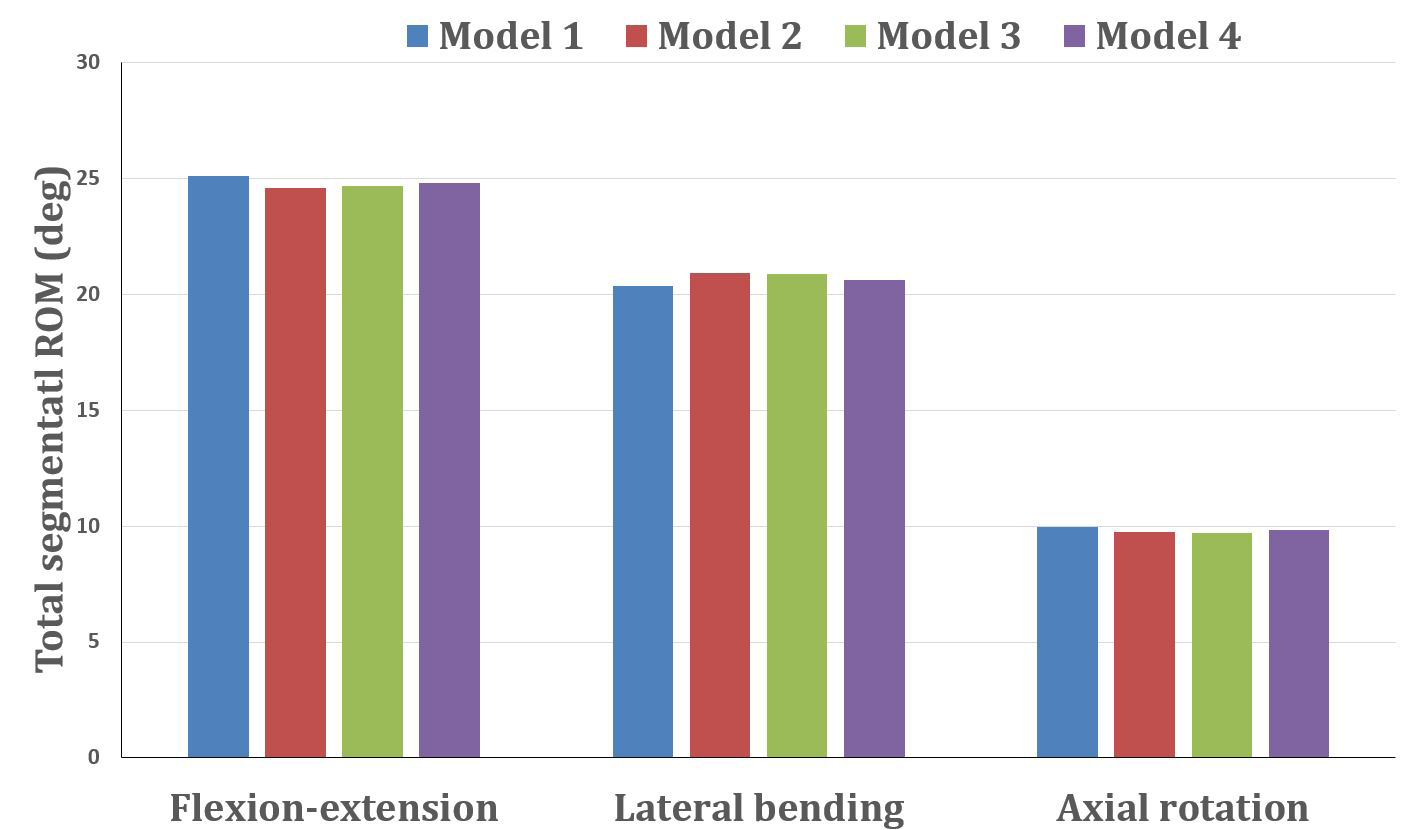

Supplement: S6 Fig — The difference between models was within 2.63%. (TIF) [file pone.0144637.s006.tif]
